# Lipid treatment

**DOI:** 10.1093/ehjcvp/pvaf009

**Published:** 2025-03-13

**Authors:** Stefan Agewall

**Affiliations:** Editor-in-Chief, Institute of Clinical Sciences, Karolinska Institute of Danderyd, Stockholm, Sweden

**Figure 1:**
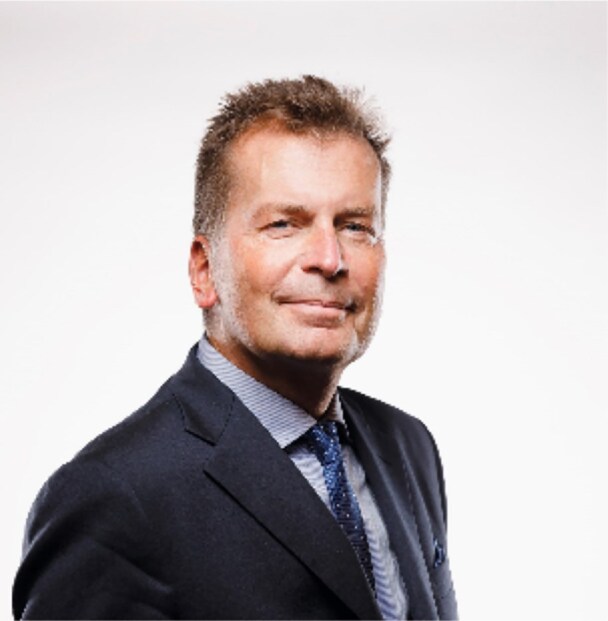


Lipid-lowering drugs (LLDs) and antihypertensive drugs are cornerstones in the treatment of atherosclerotic aortic disease.^1^^,^^2^ Dr Zhou and co-workers from China aimed to assess the impact of (LLDs) on the risk of aortic diseases. Mendelian randomization (MR) was utilized to analyse data from 500 000 participants in the UK Biobank to evaluate the effects of statins and PCSK9 inhibitors (PCSK9i on the risks of thoracic aortic aneurysm, abdominal aortic aneurysm, and aortic dissection using genetic variants as proxies). The authors reported that LLDs, particularly statins and PCSK9i, significantly protect against aortic diseases, providing a scientific basis for preventing and treating aortic diseases. The underlying mechanisms of aortic diseases likely involve an interplay between genetic predispositions and these acquired risk factors.^2^

Calcific aortic stenosis (AS) is the most common valvular pathology in the developed world. Large observational and MR studies have demonstrated a strong association between both elevated LDL cholesterol (LDL-c) and triglycerides (TG) with risk of AS, although randomized trials showed no benefit of statins for AS. Yet, despite its rising prevalence there remains no evidence-based medical therapy. Concordantly registry studies have demonstrated increased risk of aortic valve calcification among patients with Familial Hypercholesterolaemia. However, the randomized trials SEAS, SALTIRE, and ASTRONOMER consistently showed no benefit of statins for AS.^3^ Dr Ciofani and co-workers from Sydney in Australia used a drug-target MR approach to investigate the genetically predicted effect of lipid-lowering therapies on risk of AS. They report that genetically proxied lipid-lowering therapies are significantly associated with reduced risk of AS. Early initiation and sustained administration of lipid-lowering therapies may prevent AS progression and warrants further research in the clinical trial setting even though the optimal treatment is not known.^4–6^

Lipid-lowering treatment is a standard after acute myocardial infarction.^7–10^ Dr Patti *et al.* from Italy evaluated the impact on cardiovascular outcome of the systematic introduction of a personalized strike early and strong (SES) approach for lipid-lowering therapy (LLT) in patients admitted for (MI). The authors retrospectively analysed data from 500 consecutive patients hospitalized across three periods. The systematic introduction of a personalized, SES strategy for LLT in patients with acute MI led to greater achievement of LDL-C goal and lower risk of major adverse cardiovascular events (MACE) at 1 year vs. the stepwise approach.

Randomized controlled trials have demonstrated the kidney protective effects of SGLT2 inhibitors.^11–13^ Dr Kaneko and his co-workers from Japan wanted to investigate the clinical significance of the modification of the kidney protective effects of SGLT2 inhibitors by baseline body mass index (BMI) compared to Dipeptidyl Peptidase IV (DPP4) inhibitors.

Their epidemiological study substantiated the improved kidney outcomes in the SGLT2 inhibitor users compared to the DPP4 inhibitor users across a wide range of BMI, which was pronounced for individuals with higher BMI.

The consideration of liver disease in clinical medicine often leads to a decision to withhold LLDs from affected patients, which may harm patients by depriving them of well-established benefits of lipid-lowering therapy on morbidity and mortality related to cardiovascular disease (CVD).^14^ The aim of a review paper by Dr Drexel *et al.* was to examine the evidence for both the benefits and risks of LLDs on the liver. Given that this risk-benefit balance varies according to the pharmacological properties of different classes of LLDs. The goal of the article is to provide guidance on therapy for patient with hepatic impairments, specifically to identify who will or will not benefit from lipid-lowering treatment in relation to CVD outcomes.

Both glucagon-like peptide-1 receptor agonists (GLP-1 RAs) and SGLT2 inhibitors have shown reduced MACE rates among different patient groups.^15^^,^^16^ Dr Liao *et al.* from China present a meta-analysis of 26 randomized controlled trials evaluating GLP-1 RAs or SGLT2is against placebo or standard care in 151 789 ASCVD patients. The authors concluded that GLP-1 RAs show benefits in peripheral artery disease and post-acute cardiovascular events, while SGLT2 demonstrated advantages in ASCVD with comorbid chronic kidney disease. Both are effective in heart failure (HF). These findings support tailored treatment strategies for diverse ASCVD participants.

SGLT2 inhibitors improve prognosis in chronic HF, but what happens haemodynamic parameters of the heart? In a prospective, double-blind, placebo-controlled study, Dr Bogoviko and co-workers from Germany, aimed to assess whether treatment with empagliflozin 25 mg/day in patients with acute HF improves echocardiographic parameters of load, left ventricular or right ventricular function.^17^ The authors concluded that after 5 days of treatment, patients in the empagliflozin cohort showed a relevant decrease in left atrial volume.

Dr Kimura and co-workers from Japan compared aspirin monotherapy to P2Y12 inhibitor monotherapy following short or very short DAPT after complex PCI. The patients were Among 5833 patients in the 30-day main study (STOPDAPT3),^18–20^ there were 1415 patients who underwent complex PCI (aspirin group: *N* = 705, and clopidogrel group: *N* = 710). The authors concluded that in the present study, the incidence of cardiovascular events nor bleedings beyond 1 month and up to 1 year was not different between aspirin monotherapy and clopidogrel monotherapy.
